# A 2-Year, Phase IV, Multicentre, Observational Study of Ranibizumab 0.5 mg in Patients with Neovascular Age-Related Macular Degeneration in Routine Clinical Practice: The EPICOHORT Study

**DOI:** 10.1155/2014/857148

**Published:** 2014-04-28

**Authors:** Sergio Pagliarini, Stephen Beatty, Blandina Lipkova, Eduardo Perez-Salvador Garcia, Stefaan Reynders, Margarita Gekkieva, Abdelkader Si Bouazza, Stefan Pilz

**Affiliations:** ^1^Department of Ophthalmology, University Hospitals Coventry & Warwickshire NHS Trust, Coventry, West Midlands CV2 2DX, UK; ^2^Institute of Eye Surgery and Macular Pigment Research Group, Whitfield Eye Clinic, Waterford, Ireland; ^3^Department of Ophthalmology, Faculty Hospital, Vojtecha Spanyola 43, 012 07 Zilina, Slovakia; ^4^Hospital Provincial Divino Valles, Avenida Islas Baleares 1, 09006 Burgos, Spain; ^5^Oogcentrum Oostende, Koninginnelaan 54, 8400 Oostende, Belgium; ^6^Novartis Pharma AG, CH-4002 Basel, Switzerland

## Abstract

*Purpose*. To assess the safety profile of ranibizumab 0.5 mg in patients with neovascular age-related macular degeneration (nAMD) in routine clinical practice. *Methods*. This 2-year, multicentre, observational study was conducted to capture real-world early practice and outcomes across Europe, shortly after European licensing of ranibizumab for nAMD. Being observational in nature, the study did not impose diagnostic/therapeutic interventions/visit schedule. Patients were to be treated as per the EU summary of product characteristics (SmPC) in effect during the study. Key outcome measures were incidence of selected adverse events (AEs), treatment exposure, bilateral treatment, compliance to the EU SmPC, and best-corrected visual acuity (BCVA) over 2 years. *Results*. 755 of 770 patients received treatment. Ranibizumab was generally well tolerated with low incidence of selected AEs (0%–1.9%). Patients received 6.2 (mean) injections and 133 patients received bilateral treatment over 2 years. Protocol deviation to treatment compliance was reported in majority of patients. The observed decline in mean BCVA (Month 12, +1.5; Month 24, –1.3 letters) may be associated with undertreatment as suggested by BCVA subgroup analysis. *Conclusion*. The EPICOHORT study conducted in routine clinical practice reinforces the well-established safety profile of ranibizumab in nAMD. In early European practice it appeared that the nAMD patients were undertreated.

## 1. Introduction

Ranibizumab (Lucentis, Novartis Pharma AG, Basel, Switzerland, and Genentech Inc., South San Francisco, CA, USA) is a humanised monoclonal antibody Fab fragment, specifically designed for ocular use, that binds with high affinity to and inhibits multiple biologically active isoforms of vascular endothelial growth factor A (VEGF-A; [1]). Ranibizumab was approved for the treatment of neovascular age-related macular degeneration (nAMD) in many countries worldwide as of 2006. The efficacy and safety profile of ranibizumab in nAMD is well established based on the results of several randomised clinical trials (RCTs) conducted on approximately ≥7500 patients [2–11]. The prospective extension studies, SECURE and HORIZON, have also established the long-term safety profile of ranibizumab 0.5 mg over a follow-up period of 24 months [12, 13].

RCTs are by nature conducted in a controlled setting, have stringent inclusion and exclusion criteria, and mostly include treatment-naïve patients. Consequently, it is of interest to translate RCT results into real-world clinical practice. Therefore, there is a need for observational studies conducted on a large and diverse patient population to further evaluate the potential risks of serious but infrequent adverse events (AEs) and the efficacy of ranibizumab in a real-world setting. Evidence to establish the safety profile of ranibizumab in routine clinical practice is growing with small clinical studies as well as with the large on-going LUMINOUS study [14–20].

The EPICOHORT study was designed, as part of the on-going postmarketing commitment of Novartis Pharma AG (Basel, Switzerland) to the European Medicines Agency (EMA). The EPICOHORT was a postapproval study designed to assess the safety profile, treatment patterns, and efficacy of ranibizumab in real-life clinical setting/routine clinical practice. This 2-year study was conducted between September 30, 2008, and November 15, 2011, in a cohort of European ophthalmology clinics that had, by EMA request, not previously participated in clinical studies with ranibizumab. Ranibizumab treatment was based on investigator's judgment and guided by EU SmPC in effect during the time of study limited by their daily practice. More specifically, up until September 19, 2011, the EU SmPC retreatment criteria were based on a loss of visual acuity (VA). All clinical assessments were performed at the discretion of the study investigator as part of his/her current practice to reflect the real-life practice in EU ophthalmology clinics. This paper reports the findings from this 2-year, phase IV, multicentre, observational study conducted in a real-world setting.

## 2. Materials and Methods

### 2.1. Study Design

This was a 2-year, multicentre, phase IV, open-label, observational, noncomparative cohort study on patients with nAMD from 54 European clinical centres that had not participated in clinical studies with ranibizumab. The study was conducted in accordance with the International Conference on Harmonisation Tripartite Guidelines for Good Clinical Practice, with applicable local regulations and ethical principles laid down in the Declaration of Helsinki. The study protocol was reviewed and approved by the Independent Ethics Committee or Institutional Review Board of each centre. Written informed consent was obtained from each participating patient before enrolment.

### 2.2. Patients

The outpatient study population consisted of patients diagnosed with choroidal neovascularisation (CNV) secondary to AMD. The patients were to be treated with ranibizumab at study entry.

### 2.3. Treatment

Due to the observational nature of the study, the study protocol did not impose diagnostic/therapeutic interventions like optical coherence tomography (OCT) assessments or a visit schedule. Patients were treated with the commercially available intravitreal injections of ranibizumab 0.5 mg (Lucentis) as per the investigators' judgement and guided by the current EU SmPC in effect during the time of the study, During the study period (September 30, 2008, to November 15, 2011), the SmPC was updated nine times and all SmPC versions before September 2011 had the same retreatment criteria. The treatment criteria were to treat with ranibizumab 0.5 mg when patients experienced a loss of ≥5 letters in VA (Early Treatment Diabetic Retinopathy Study (ETDRS) letters or Snellen equivalent). Data were recorded by the investigator at each patient visit.

### 2.4. Study Objectives

The key objectives of the study were to describe the characteristics of nAMD patients treated with ranibizumab in European clinics; incidence of bilateral use of ranibizumab; compliance to the EU SmPC in effect during the time of the study; incidence of the following selected adverse events (AEs): endophthalmitis, retinal detachment, vitreous haemorrhage, retinal tear, traumatic cataract, uveitis, and elevated intraocular pressure (IOP); and best-corrected visual acuity (BCVA) as a clinical efficacy measure in the study population.

### 2.5. Assessments and End Points

#### 2.5.1. Patient Demographics and Other Baseline Characteristics

Demographic patient data including age, gender, race, and ethnicity were collected at the study entry. Details on medical history and current medical conditions (including ocular-related history/conditions), on-going medications, and significant nondrug therapies were also recorded, if provided by the patient. BCVA was assessed at study entry or if not done a prior VA value closest to the study entry was recorded. However, during the analysis, patients who did not have the baseline VA were excluded from the analyses.

#### 2.5.2. Treatment Exposure

The number of ranibizumab injections and the bilateral use of ranibizumab were recorded over the 2-year treatment period. Bilateral use of ranibizumab was assessed by (i) proportion of patients receiving ranibizumab treatment in both eyes at any time during the study and (ii) proportion of patients with bilateral treatment within 28 days and on the same day.

#### 2.5.3. Compliance to the Current EU SmPC

Data on compliance to the EU SmPC in terms of treatment dose, use of antimicrobial eye drops, treatment despite contraindications, interval between two treatments, and compliance to retreatment criteria, as well as reported reasons for noncompliance, were collected at every patient visit.

#### 2.5.4. Safety Assessment

Safety profile was assessed by recording the incidence of AEs and serious adverse events (SAEs) over the 2-year assessment period. AEs were assessed by the investigator by nondirective questioning of patients at each visit (i.e., asking questions without including symptoms, so as not to influence the outcomes), voluntary reports by the patients during or between visits or through physical examination. Incidence of the selected AEs (endophthalmitis, retinal detachment, vitreous haemorrhage, retinal tear, traumatic cataract, uveitis, and elevated IOP) was assessed based on the records provided by the study investigator. The incidence was calculated on a per-patient basis over 2 years. In addition, the incidence of the selected AEs was also calculated on per-injection and per-patient-year basis for a period of 2 years.

#### 2.5.5. Best-Corrected Visual Acuity

BCVA was assessed using either the Snellen or ETDRS charts. For the analysis, the Snellen equivalent was converted to an ETDRS score. The efficacy end point was mean change in BCVA letter score from baseline to Month 24. Other efficacy end points included proportion of patients with BCVA letter score gain of ≥15 ETDRS letters and loss of <15 ETDRS letters from baseline to Month 24. An exploratory subgroup analysis on efficacy was performed based on the number and timing of initial injections. The subgroups data reported here were descriptive only and no statistical analysis was intended. The categories assessed were based on the number of injections within 90 days from baseline (<3, ≥3 injections) and number of injections within 90 days from baseline and overall injections (≥3 injections within 90 days from baseline but <10 injections overall and ≥3 injections within 90 days from baseline and ≥10 injections overall).

### 2.6. Statistical Analysis

The EPICOHORT study focused primarily on the safety profile of ranibizumab and no statistical hypothesis testing was intended. All analyses were performed on the safety set, which included patients treated with at least one dose of ranibizumab and who had at least one safety assessment after the first treatment. All analyses were conducted on the safety set except for patient disposition, which was based on the enrolled set (patients who entered into the study and for whom data were recorded). Patient characteristics were summarised using descriptive statistics. All AEs were reported based on the Medical Dictionary for Regulatory Activities (MedDRA) system organ classes and preferred terms. The incidence of AEs (ocular/nonocular) was listed based on the seriousness and relationship to the study drug/procedure. The incidence of selected AEs was calculated based on the proportion of patients with at least one AE over 2 years. The corresponding 95% (Clopper-Pearson) confidence intervals for the incidence rate were presented on a per-patient basis. Ocular and nonocular safety assessments were carried out including assessments of the second-treated eye (as applicable). The first-treated eye was the eye that received the first dose of treatment according to the date and time of treatment during the study. The other eye was considered as a fellow eye (nontreated) until the date it was treated during the study, at which it became the second-treated eye. In this paper, we mainly report the results for the first-treated eye. Compliance to the EU SmPC was summarised using descriptive statistics. All efficacy analyses were performed on the observed data of the safety set. In addition, the analysis was repeated using the last observation carried forward (LOCF) results (i.e., carrying forward the last nonmissing postbaseline results).

## 3. Results

### 3.1. Patient Disposition and Baseline Characteristics

**Figure 1:**
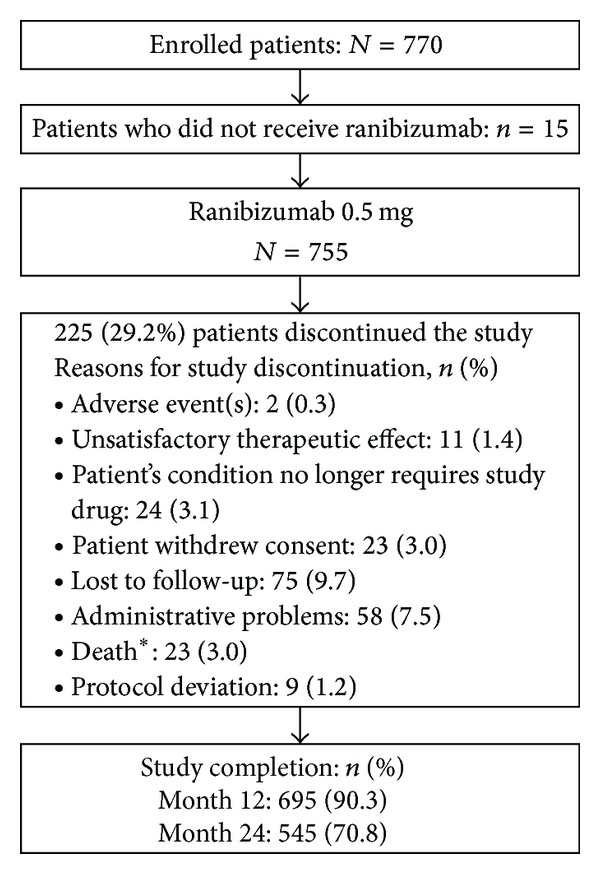
Patient disposition (all enrolled patients). *Except for one, none of the deaths were suspected to be related to the study treatment.

A total of 770 patients were enrolled in this study and the gross enrolment happened within 4 months of the study. Out of 770 enrolled patients, 755 patients received treatment. Fifteen patients did not receive any ranibizumab treatment and therefore were not included in the safety analysis. Overall, 695 (90.3%) patients and 545 (70.8%) patients completed the study in years 1 and 2, respectively. The premature study discontinuation was mainly due to “administrative reasons” (58 [7.5%]) or patients “lost to follow-up” (75 [9.7%]). Two patients (0.3%) discontinued the study due to an AE and 11 patients (1.4%) discontinued because of unsatisfactory therapeutic effect. The proportion of patients who discontinued the study and the reasons for discontinuation are shown in Figure 1. The mean (±SD) age of patients was 76.3 (±8.7) years and the majority of them were ≥65 years and Caucasian (99.2%). The mean (±SD) BCVA of the first-treated eye and the nontreated eye at baseline was 52.8 (±20.3) and 57.2 (±20.2) letters, respectively. Prior ocular medication was voluntarily reported in 270 (35.8%) patients with ranibizumab being the most frequently used medication in 251 (33.2%) patients.

### 3.2. Treatment Exposure and Compliance

#### 3.2.1. Ranibizumab Injections

A mean of 6.2 ranibizumab injections was administered during the study (first year: 4.4; second year: 1.8) over 2 years (Table 1).

#### 3.2.2. Bilateral Treatment

A mean (±SD) of 4.9 (±3.5) injections was administered in the second-treated eye over 2 years. Overall, 133 (17.6%) patients received bilateral treatment during the study, of which 87 (11.5%) patients received bilateral treatment within 28 days and 19 (2.5%) patients received bilateral treatment on the same day (Table 1).

#### 3.2.3. Compliance to the Current EU SmPC

Patients with at least one protocol deviation were recorded during the study. The majority of protocol deviations were related either to patients not self-administering antimicrobial eye drops before or after the injection (360 [47.7%]) or to minor procedural deviations (379 [50.2%]). These procedural deviations were mainly related to the patient reconsent process after the informed consent amendment and to the delay of SAE reporting (Table 2). Further analysis revealed that more than 10,000 of 13,483 evaluations performed for treatment need were in accordance with the EU SmPC requirement, and approximately half of a total of 5,368 treatments given were compliant with the EU SmPC. The reasons for noncompliance were not systematically recorded. The median of the minimum duration between 2 consecutive visits was 29 days.

### 3.3. Safety

#### 3.3.1. Selected AEs

The overall incidence of the selected AEs in the first-treated eye is shown in Table 3. Over the 2-year study period, no cases of retinal detachment or traumatic cataract were reported, whereas endophthalmitis and uveitis were reported in one (0.1%) patient each in the first-treated eye. Following bilateral treatment (second-treated eye), vitreous haemorrhage and elevated IOP were reported in one (0.1%) patient each. However, no cases of endophthalmitis, retinal detachment, retinal tears, traumatic cataract, or uveitis were reported in patients with second-treated eye. The selected AEs analysed based on per injection, per patient-year, and per treated eye are presented in Table 4.

#### 3.3.2. Serious Adverse Events

Overall, ocular SAEs (first-treated eye) were reported in 12 (1.6%) patients (Table 5). Except for retinal haemorrhage (4 patients, 0.5%) and retinal pigment epithelial (RPE) tear (2 patients, 0.3%), all SAEs were reported in one patient each. Ocular SAEs suspected to be related to the study drug and/or treatment procedure were reported in 6 (0.8%) patients. Ocular SAEs suspected to be related to the study drug were reported in 4 (0.5%) patients. Endophthalmitis and retinal haemorrhage were reported in 1 (0.1%) patient each, both suspected by the investigator to be related to the study drug and treatment procedure.

Nonocular SAEs were reported in 89 (11.8%) patients. Most frequent nonocular SAEs were cerebrovascular accident, chronic obstructive pulmonary disease, and hypertension, each reported in 5 (0.7%) patients (Table 5). Nonocular SAEs suspected to be related to the study drug and/or treatment procedure were reported in 6 (0.8%) patients. Nonocular SAEs suspected to be related to study drug were reported in 5 (0.7%) patients.

There were 23 (3%) deaths reported during the study. Except for one, none of the deaths were suspected to be related to the study treatment. For this one patient, who had a medical history of hypertension and myocardial ischaemia, the cause of death was stroke and the event was suspected to be related to the study drug and treatment procedure, by the investigator.

#### 3.3.3. Adverse Events

Ocular AEs (first-treated eye) were reported in 255 (33.8%) patients. The most frequently reported ocular AEs were conjunctival haemorrhage (58 [7.7%]), eye irritation (25 [3.3%]), and cataract (24 [3.2%]; Table 6). RPE tear (7 [0.9%]) was the most frequently reported ocular AE suspected by the investigator to be related to the study drug. Nonocular AEs were reported in 236 (31.3%) patients (Table 6). The most frequently reported nonocular AEs were hypertension (19 [2.5%]) and influenza (17 [2.3%]). Hypertension (4 [0.5%]) was the most frequent nonocular AE suspected by the investigator to be related to the study drug.

### 3.4. Efficacy

#### 3.4.1. Best-Corrected Visual Acuity

**Figure 2:**
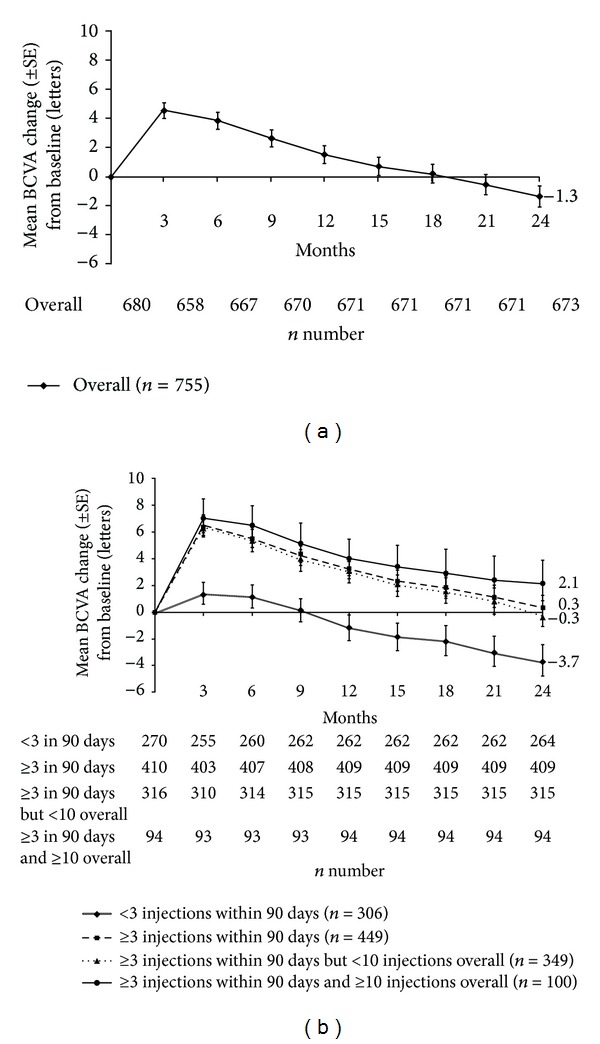
(a) Overall mean change in BCVA over time in the first-treated eye and (b) mean change in BCVA overtime based on the number of injections in the first-treated eye (safety set, LOCF). Safety set was defined as all those patients who were treated with at least one dose of ranibizumab and who had at least one safety assessment after study entry and no protocol deviations that warranted exclusion. Baseline VA results were recorded for 680 patients. Month 24 results were evaluated for 673 patients using the LOCF approach. BCVA: best-corrected visual acuity; LOCF: last observation carried forward.

Ranibizumab treatment led to BCVA (mean [±SE]) improvements at Month 3 (+4.5 [0.52] letters) and then to a continuous decline in BCVA until Month 24. At Months 12 and 24, the mean change (±SE) in BCVA from baseline was +1.5 (0.61) letters and −1.3 (0.72) letters, respectively (Figure 2(a)). A subgroup analysis on BCVA based on the number and timing of initial injections demonstrated that patients receiving <3 injections within the first 90 days (*n* = 306) lost 3.7 letters, whereas patients receiving ≥3 injections within the first 90 days (*n* = 449) gained 0.3 letters at Month 24. In addition, patients who received ≥3 injections within 90 days but <10 injections overall (*n* = 349) lost 0.3 letters, while patients who received ≥3 injections within 90 days and ≥10 injections overall (*n* = 100) gained 2.1 letters at Month 24 (Figure 2(b)).

## 4. Discussion

The EPICOHORT study conducted in a routine clinical practice in Europe demonstrated that ranibizumab 0.5 mg therapy was well tolerated in patients with nAMD, with no new ocular or nonocular safety findings observed over the 2-year study period. The broad involvement of European clinics across 54 centres and an observational design of the study (i.e., no treatment protocol) reflect the real-life practice in European ophthalmology clinics.

In the EPICOHORT study, the incidence of selected AEs was low (0%–1.9%) and similar to that observed in RCTs with pro re nata (PRN) regimen in nAMD patients [12, 13, 21–23]. This indicates that the safety profile of ranibizumab in clinical practice is not substantially different from what was reported in RCTs with PRN regimen. However, these results need to be interpreted with caution due to differences in comorbidities, study design, and treatment criteria used in these trials.

Of a total of 4711 ranibizumab injections administered during the study, only one case of endophthalmitis (0.0002/injection) was reported. In this case, the patient received 40 intravitreal ranibizumab injections prior to the event (20 in the first-treated eye and 20 in the second-treated eye); the first-treated eye with endophthalmitis responded to ranibizumab treatment and the patient completed the study with a gain of 3 letters in that eye. Although 19 patients received bilateral treatment on the same day, no cases of endophthalmitis were reported in these patients. In addition, there were no unexpected safety findings in both the bilateral treatment categories whether administered on the same day (*n* = 19) or within 28 days (*n* = 87). Overall, the incidence of endophthalmitis and other selected AEs observed in this study is comparable to that observed in RCTs conducted in patients with nAMD. Additionally, the incidence of selected AEs observed in this study was also comparable to those observed in the LUMINOUS retrospective study, which was also conducted in a real-life setting [17–20].

The incidence of ocular and nonocular AEs and SAEs over the 2-year study period was low and consistent with those observed in RCTs using PRN regimen in patients with nAMD [12, 13, 21–23]. Of the 23 (3.0%) deaths reported during the study, except for one, none of the other deaths were suspected to be related to the study drug or treatment procedure by the investigator. For this one patient, the cause of death was stroke and the patient had a medical history of hypertension and myocardial ischemia. Overall, there were no new ocular and nonocular safety findings observed in the EPICOHORT study, and the safety profile was consistent with the well-established safety profile of ranibizumab in nAMD.

Over the 2-year study period, at least one protocol deviation was reported in majority of the patients. It may be inferred that due to the observational nature of this study and especially since the clinics chosen were participating for the first time in a ranibizumab clinical trial, the standard procedure of ocular injections (as per the EU label) may not be consistently followed. This is supported by the observation that the majority of protocol deviations were due to omission of antimicrobial eye drops before or after injection (47.7%). Despite this noncompliance to the EU SmPC and protocol deviations, the overall incidence of endophthalmitis (0.1%; 0.0002/injection) was low and within the range as observed in RCTs with PRN regimen [12, 13, 21–23]. In addition, only two patients prematurely discontinued the study due to an AE, with the reasons being mainly “administrative reasons” (7.5%) or patients “lost to follow-up” (9.7%).

In the current study, a mean BCVA gain of +4.5 letters from baseline was observed at Month 3 followed by a decline over 24 months. A subgroup analysis of BCVA improvement based on the number of injections showed that more than 1/3 (*n* = 306) of the overall study population received <3 injections within 90 days, although the EU label in effect during the time of study recommended three initial monthly loading injections. This may be because over 1/3 of the overall population (*n* = 270 [35.8%]) was not naïve to nAMD treatment and had been pretreated, mainly with ranibizumab (*n* = 251 [33.2%]). These pretreated patients may not have required the three monthly loading injections of ranibizumab within 3 months of recruitment and were less likely to have improvements in BCVA than the treatment-naïve patients. This may explain the lack of mean BCVA improvement in patients receiving <3 injections within 90 days (*n* = 306; Figure 2(b)). On the other hand, patients who received ≥3 injections within 90 days (*n* = 449) of recruitment improved their BCVA, of which 100 patients who were treated with ≥10 injections over 24 months showed an improvement of 2.1 letters.

Overall, the mean number of injections was low (6.2) and potentially some patients may have been undertreated during the study. The possible undertreatment may be due to the observational nature of the study design (without a scope for specific retreatment criteria other than that in the product label, a standard diagnostic/therapeutic intervention or a visit schedule) and factors related to local practice (e.g., reimbursement issues, patient follow-up). Additionally, for a major part of the study period (i.e., until September 2011) the patients were treated only if they experienced a loss of ≥5 letters as per the current EU SmPC at the time of study. This may have led to retreatment decisions reactive to loss of VA rather than proactive that aim at detecting the early signs of disease activity before actual VA loss. In contrast to this study, a more recent study that has used PRN dosing regimen using both VA and OCT retreatment criteria with monthly monitoring showed VA outcomes comparable to that observed with monthly ranibizumab treatment [23]. Thus, detecting the early signs of anatomical changes and treating the disease activity promptly before actual VA loss along with adequate monitoring may help to achieve better visual gains.

In summary, ranibizumab treatment was generally well tolerated in patients with CNV secondary to AMD in routine clinical practice in European clinics that had not previously participated in clinical trials with ranibizumab. The incidence of selected AEs was low, and no new ocular or nonocular safety findings were observed in this study. Noncompliance to prior antibiotic application did not seem to have any effect on the incidence of endophthalmitis. The recommendation for the patient self-administering antimicrobial drops, four times daily for 3 days before and after injection, is now removed in the current EU label (January 2014). Further studies supporting ranibizumab use in clinical practice are on-going. The on-going LUMINOUS study (ClinicalTrials.gov [NCT01318941]), which is successfully enrolling patients worldwide, will help to provide further evidence on the long-term safety, effectiveness, treatment patterns, and quality-of-life outcomes of ranibizumab treatment in routine clinical practice.

## Figures and Tables

**Table 1:** Treatment exposure and the incidence of bilateral treatment (safety set).

	Ranibizumab 0.5 mg total (*N* = 755)
Number of injections in the first-treated eye	
Year 1	3306
Mean (SD)	4.4 (2.3)
Overall (24 months)	4711
Mean (SD)	6.2 (4.3)
Patients with bilateral treatment over 24 months, *n* (%)	133 (17.6)
Patients with bilateral treatment within 28 days, *n* (%)	87 (11.5)

Safety set was defined as all those patients who were treated with at least one dose of ranibizumab and who had at least one safety assessment after study entry and no protocol deviations that warranted exclusion.

The total number of injections is number of injections received since the start of the study in the specific eye.

Patients with bilateral treatment represent the patients who received treatment in both eyes.

SD: standard deviation.

**Table 2:** Protocol deviations with respect to treatment compliance over 2 years.

Protocol deviation	Ranibizumab 0.5 mg total (*N* = 755)
At least one protocol deviation*, *n* (%)	
Injection of more than 0.5 mg (overdose)	1 (0.1)
Minor procedural deviations without obvious impact on the safety of the patient	379 (50.2)
No single use of vial (multiple injections)	40 (5.3)
Patient did not self-administer antimicrobial drops (4 times daily for 3 days) before or after the injection	360 (47.7)
Patient has no CNV secondary to AMD	2 (0.3)
Overall instances of each protocol deviation^†^, *n*/*N*	
Injection of more than 0.5 mg (overdose)	1/1
Minor procedural deviations without obvious impact on the safety of the patient	575/379
No single use of vial (multiple injections)	40/40
Patient did not self-administer antimicrobial drops (4 times daily for 3 days) before or after the injection	2242/360
Patient has no CNV secondary to AMD	2/2

*At least one section only counts a protocol deviation once per patient.

^†^Overall instance section reports all instances of each protocol deviation (*n*) and the total number of patients involved in those instances (*N*).

AMD: age-related macular degeneration; CNV: choroidal neovascularisation.

**Table 3:** Incidence of selected AEs over 2 years (safety set, first-treated eye).

Preferred term	Ranibizumab 0.5 mg total, *n* (%) (*N* = 755)	95% CI* (LL%, UL%)
Total	21 (2.8)	(1.7, 4.2)
Intraocular pressure increased	14 (1.9)	(1.0, 3.1)
Vitreous haemorrhage	3 (0.4)	(0.1, 1.2)
Retinal tears	2 (0.3)	(0.0, 1.0)
Endophthalmitis	1 (0.1)	(0.0, 0.7)
Uveitis	1 (0.1)	(0.0, 0.7)
Cataract traumatic	0	(0.0, 0.5)
Retinal detachment	0	(0.0, 0.5)

Safety set was defined as all those patients who were treated with at least one dose of ranibizumab and who had at least one safety assessment after study entry and no protocol deviations that warranted exclusion.

*Exact Binomial (Clopper-Pearson).

Special event-preferred terms are presented in the descending order of frequency.

AEs occurring only during the safety observation period are included.

A patient with multiple occurrences of an AE is counted only once in the preferred term category.

AE: adverse event; CI: confidence interval; LL: lower limit; UL: upper limit.

**Table 4:** Selected AEs over 2 years: per injection, per patient-year (first-treated eye), and per treated eye (safety set).

Preferred term	Ranibizumab 0.5 mg total (*N* = 755)
AEs per injection*	
Total number of injections	4711
Intraocular pressure increased	40 (0.0085)
Vitreous haemorrhage	3 (0.0006)
Retinal tear(s)	2 (0.0004)
Endophthalmitis	1 (0.0002)
Uveitis	1 (0.0002)
Cataract traumatic	0
Retinal detachment	0
AEs per patient-year^†^	
Total number of years	1361
Intraocular pressure increased	40 (0.0294)
Vitreous haemorrhage	3 (0.0022)
Retinal tear(s)	2 (0.0015)
Endophthalmitis	1 (0.0007)
Uveitis	1 (0.0007)
Cataract traumatic	0
Retinal detachment	0
AEs per treated eye^‡^	
Total number of treated eyes	888
Intraocular pressure increased	41 (0.0462)
Vitreous haemorrhage	5 (0.0056)
Retinal tear(s)	2 (0.0023)
Endophthalmitis	1 (0.0011)
Uveitis	1 (0.0011)
Cataract traumatic	0
Retinal detachment	0

Safety set was defined as all those patients who were treated with at least one dose of ranibizumab and who had at least one safety assessment after study entry and no protocol deviations that warranted exclusion.

*Number of AEs (rate per injection), where rate per injection is calculated as the number of events/total number of injections.

^†^Number of AEs (rate per year), where rate per patient-year of safety observation is calculated as the number of events/total number of patient-years.

^‡^Number of AEs (rate per eye), where rate per treated eye is calculated as the number of events/total number of treated eyes.

Preferred terms are presented in the descending order of frequency.

All occurrences of the AE during the safety observation period are included in the AE category (including multiple occurrences per patient).

AE: adverse event.

**Table 5:** Patients with ocular SAEs and frequent nonocular SAEs (≥3 patients) over 2 years (safety set, first-treated eye).

Preferred term	Ranibizumab 0.5 mg total, *n* (%) (*N* = 755)	95% CI* (LL%, UL%)
Ocular SAEs		
Total	12 (1.6)	(0.8, 2.8)
Retinal haemorrhage	4 (0.5)	(0.1, 1.4)
Retinal pigment epithelial tear	2 (0.3)	(0.0, 1.0)
Angle closure glaucoma	1 (0.1)	(0.0, 0.7)
Endophthalmitis	1 (0.1)	(0.0, 0.7)
Glaucoma	1 (0.1)	(0.0, 0.7)
Intraocular pressure increased	1 (0.1)	(0.0, 0.7)
Macular hole	1 (0.1)	(0.0, 0.7)
Open angle glaucoma	1 (0.1)	(0.0, 0.7)
Optic neuritis	1 (0.1)	(0.0, 0.7)
Visual acuity reduced	1 (0.1)	(0.0, 0.7)
Vitreous haemorrhage	1 (0.1)^†^	(0.0, 0.7)
Nonocular SAEs		
Total	89 (11.8)^‡^	(9.6, 14.3)
Cerebrovascular accident	5 (0.7)	(0.2, 1.5)
Chronic obstructive pulmonary disease	5 (0.7)	(0.2, 1.5)
Hypertension	5 (0.7)	(0.2, 1.5)
Pneumonia	4 (0.5)	(0.1, 1.4)
Cardiac failure	3 (0.4)	(0.1, 1.2)
Colon cancer	3 (0.4)	(0.1, 1.2)
Depression	3 (0.4)	(0.1, 1.2)
Femur fracture	3 (0.4)	(0.1, 1.2)

Safety set was defined as all those patients who were treated with at least one dose of ranibizumab and who had at least one safety assessment after study entry and no protocol deviations that warranted exclusion.

*Exact Binomial (Clopper-Pearson).

^†^Patient discontinued the treatment due to SAE.

^‡^17 patients discontinued the treatment due to nonocular SAE.

SAEs occurring only during the safety observation period are included.

Preferred terms are presented in the descending order of frequency.

A patient with multiple occurrences of an AE is counted only once in the preferred term category.

A patient with multiple AEs is counted only once in the total row.

AE: adverse event; CI: confidence interval; LL: lower limit; SAE: serious adverse event; UL: upper limit.

**Table 6:** Patients with frequent (≥1%) ocular and nonocular AEs over 2 years (safety set, first-treated eye).

Preferred term	Ranibizumab 0.5 mg total, *n* (%) (*N* = 755)	95% CI* (LL%, UL%)
Ocular AEs		
Total	255 (33.8)	(30.4, 37.3)
Conjunctival haemorrhage	58 (7.7)	(5.9, 9.8)
Eye irritation	25 (3.3)	(2.2, 4.8)
Cataract	24 (3.2)	(2.0, 4.7)
Conjunctivitis	22 (2.9)	(1.8, 4.4)
Eye pain	21 (2.8)	(1.7, 4.2)
Vitreous floaters	18 (2.4)	(1.4, 3.7)
Retinal haemorrhage	17 (2.3)	(1.3, 3.6)
Intraocular pressure increased	14 (1.9)	(1.0, 3.1)
Ocular hypertension	14 (1.9)	(1.0, 3.1)
Conjunctivitis allergic	10 (1.3)	(0.6, 2.4)
Injection site discharge	10 (1.3)	(0.6, 2.4)
Retinal pigment epithelial tear	10 (1.3)	(0.6, 2.4)
Blepharitis	9 (1.2)	(0.5, 2.3)
Glaucoma	8 (1.1)	(0.5, 2.1)
Posterior capsule opacification	8 (1.1)	(0.5, 2.1)
Nonocular AEs		
Total	236 (31.3)	(28.0, 34.7)
Hypertension	19 (2.5)	(1.5, 3.9)
Influenza	17 (2.3)	(1.3, 3.6)
Diabetes mellitus	10 (1.3)	(0.6, 2.4)
Nasopharyngitis	10 (1.3)	(0.6, 2.4)
Urinary tract infection	10 (1.3)	(0.6, 2.4)
Hypercholesterolemia	9 (1.2)	(0.5, 2.3)

Safety set was defined as all those patients who were treated with at least one dose of ranibizumab and who had at least one safety assessment after study entry and no protocol deviations that warranted exclusion.

*Exact Binomial (Clopper-Pearson).

AEs occurring only during the safety observation period are included.

Preferred terms are presented in the descending order of frequency.

A patient with multiple occurrences of an AE is counted only once in the preferred term category.

A patient with multiple AEs is counted only once in the total row.

AE: adverse event; CI: confidence interval; LL: lower limit; UL: upper limit.
